# Interleukin-37 is increased in ankylosing spondylitis patients and associated with disease activity

**DOI:** 10.1186/s12967-015-0394-3

**Published:** 2015-01-28

**Authors:** Bingni Chen, Kunzhao Huang, Liang Ye, Yanqun Li, Jiawei Zhang, Jinshun Zhang, Xinmin Fan, Xiaokai Liu, Li Li, Jinxia Sun, Jing Du, Zhong Huang

**Affiliations:** Biological therapy institute, Shenzhen University School of Medicine, Shenzhen, 518060 Guangdong China; Department of Pathogen biology and immunology, Shenzhen University School of Medicine, Shenzhen, 518060 China; Shenzhen City Shenzhen University Immunodiagnostic Technology Platforms, Shenzhen, 518060 China; Department of pathology, Shenzhen University School of Medicine, Shenzhen, 518060 China; Department of Laboratory Medicine, Peking University Shenzhen Hospital, Shenzhen, 518036 Guangdong China

**Keywords:** Interleukin-37, Ankylosing spondylitis, Peripheral blood mononuclear cells, Tumor necrosis factor-α, Interleukin-17, Interleukin-6, Interleukin-23

## Abstract

**Background:**

Interleukin-37 (IL-37) has been known to play an immunosuppressive role in various inflammatory disorders, but whether it participates in the regulation of pathogenesis of ankylosing spondylitis (AS) has not been investigated. Here, we examined the serum levels of IL-37 and its clinical association in AS, and explored the anti-inflammatory effects of IL-37 on peripheral blood mononuclear cells (PBMCs) from AS patients.

**Methods:**

The mRNA levels of IL-37, TNF-α, IL-6, IL-17, and IL-23 in PBMCs and their serum concentrations from 46 AS patients were examined by real-time polymerase chain reaction (RT-PCR) and enzyme-linked immunoassay (ELISA), respectively. The correlations between serum IL-37 levels with disease activity, laboratory values and pro-inflammatory cytokines in AS were analyzed by Spearman correlation test. PBMCs from 46 AS patients were stimulated with recombinant IL-37 protein, expressions of TNF-α, IL-6, IL-17 and IL-23 were determined by RT-PCR and ELISA.

**Results:**

Compared to healthy controls (HC), AS patients and active AS patients showed higher levels of IL-37 in PBMCs and serum respectively. Strikingly, serum IL-37 levels were higher in AS patients with osteoporosis than those without. Serum levels of IL-37 were correlated with laboratory values as well as TNF-α, IL-6 and IL-17, but not IL-23 in patients with AS. The productions of pro-inflammatory cytokines such as TNF-α, IL-6, IL-17, IL-23 in PBMCs from AS patients were obviously attenuated after recombinant IL-37 stimulation, but not in the HC.

**Conclusion:**

The higher levels of IL-37 were found in AS patients, which were correlated with disease activity and AS related pro-inflammatory cytokines. More importantly, IL-37 inhibits the expressions of the pro-inflammatory cytokines from PBMCs in AS patients, indicating the potential anti-inflammatory role of IL-37 in AS.

**Electronic supplementary material:**

The online version of this article (doi:10.1186/s12967-015-0394-3) contains supplementary material, which is available to authorized users.

## Background

Ankylosing spondylitis (AS) is a prevalent chronic inflammatory disease characterized by chronic inflammation in the axial skeleton and peripheral joints respectively and leading to bone erosion, which seriously influences the quality life of patients [1]. Several studies have suggested that major histocompatibility complex class I (MHC I) might affect susceptibility to AS, 90-95% of patients with AS are Human Leukocyte Antigen (HLA) B27 positive [2,3]. The onset, pathogenic process and severity of AS are depended on the degrees of inflammation in the disease [4,5].

Although the etiology of AS is unclear, accumulating evidences have underlined that the levels of pro-inflammatory cytokines (TNF-α, IL-6, IL-17 and IL-23) were significantly increased in the peripheral blood of AS patients [6-12]. Clinical trials suggested that blocking these cytokines could partly relieve inflammatory symptoms of AS and also appears to reduce disease severity [13-15]. Recent studies have indicated that IL-37 down-regulated the expressions of pro-inflammatory cytokines in chronic inflammatory diseases such as system lupus erythematosus (SLE) [16], rheumatoid arthritis (RA) [17] and inflammatory bowel disease [18], suggesting IL-37 might abrogate pro-inflammatory cytokines productions to reduce inflammatory responses in AS.

IL-37, belongs to the members of the IL-1 family, has been described as an anti-inflammatory cytokine in several inflammatory diseases [19-21]. IL-37 is highly concentrated in the testis, thymus and uterus, and can be induced in various types of cells such as peripheral blood mononuclear cells (PBMCs), epithelial cells, dendritic cells, monocytes and keratinocytes [20-22]. Up-regulated expressions of IL-37 in serum have been reported in many inflammation-related disorders, such as systemic lupus erthymatosus (SLE) [16], rheumatoid arthritis (RA) [17] and acute coronary syndrome [23]. *In vitro,* IL-37 has been demonstrated to effectively abrogate the expressions of pro-inflammatory cytokines in several cell types, including PBMCs [20-23]. *In vivo,* IL-37 reduced the inflammatory responses and clinical symptoms of cerebral ischemia, myocardial ischaemia/reperfusion injury, psoriasis, and asthma in mouse models [24-29]. Our published studies showed that IL-37 may play a negative feedback mechanism to restrain the inflammatory reaction in SLE [16] and Graves’ Disease (GD) [30]. However, the information related to the expression and function of IL-37 in AS is still lacking.

Here, we investigated the expression of IL-37 in serum and PBMCs of patients with AS, and correlations of serum IL-37 levels with disease activity, complications, laboratory parameters and pro-inflammatory cytokines in AS. We further studied the function of IL-37 in AS by using recombinant IL-37 to treat the PBMCs from AS patients.

## Materials and methods

### Patients and controls

Forty-six AS patients from Peking University Shenzhen Hospital, Shenzhen, People’s Republic of China were invited to enroll in our research. All AS patients were individually diagnosed according to modified New York Criteria [31]. Thirty-five age-and sex-matched volunteers were recruited from Peking University Shenzhen Hospital as healthy controls (HC). We excluded other rheumatic diseases, infections or malignant tumors from the study. Clinical data from each patient like age, sex, disease duration, erythrocyte sedimentation rate (ESR), C-reactive protein (CRP), platelet (PLT) and current medications were recorded (Table 1). AS disease activity was identified by Bath Ankylosing Spondylitis Disease Activity Index (BASDAI) score [32]. BASDAI score ≥ 4 was defined as active AS [33]. The research was approved by the regional ethics committee in Peking University Shenzhen Hospital. Written informed consents were obtained from all participants.

**Table 1:** **Clinical and laboratory characteristics of the AS patients and controls**

**Characteristics**	**Active AS (n = 25)**	**Inactive AS (n = 21)**	**AS (n = 46)**	**HCs (n = 35)**
Age in years (mean)	31.2 ± 11.96	28.7 ± 7.52	30.0 ± 10.26	30.3 ± 8.87
Sex, no. Male/no. Female	21/4	13/8	34/12	26/9
HlA-B27, percentage positive (%)	23 (92)	18 (86)	5 (89)	-
Disease duration (years)	6.3 ± 4.25	5.6 ± 5.47	6.0 ± 4.76	-
Osteoporosis n (%)	18 (72)	6 (29)	24 (52)	-
Peptic ulcer n (%)	7 (28)	1 (5)	8 (17)	-
Liver dysfunction n (%)	7 (28)	10 (48)	17 (37)	-
Intestinal tuberculosis n (%)	-	1 (5)	1 (2)	-
Leukocytosis n (%)	7 (28)	-	7 (15)	-
Kidney dysfunction n (%)	4 (16)	7 (33)	11 (24)	-
Polycythemia n (%)	5 (20)	3 (14)	8 (17)	-
Hyperlipidemia n (%)	1 (4)	-	1 (2)	-
Adult still disease n (%)	1 (4)	-	1 (2)	-
CRP in mg/L (median)	15.8 ± 4.51	6.2 ± 4.31	11.4 ± 6.56	-
ESR in mm/h (median)	30.8 ± 14.45	8.3 ± 4.08	20.5 ± 15.87	-
ALT in U/L (median)	28.7 ± 21.19	31.4 ± 21.79	30.0 ± 21.50	-
PLT 10^9^/L (median)	317.4 ± 96.42	247.6 ± 61.53	285.5 ± 90.49	-
RBC in L (median)	5.1 ± 0.56	4.9 ± 0.57	5.0 ± 0.57	-
BASDAI	5.8 ± 0.90	2.9 ± 0.70	4.5 ± 1.66	-

Except where otherwise indicated, values are expressed as mean ± standard deviation. There were no significant differences between patients with AS and healthy donors in terms of age and sex; BASDAI, (range 0–10); The normal range are 0 ~ 15 mm/h for ESR; 0 ~ 5 mg/L for CRP; 4.0 ~ 5.5 × 10^12^/L for RBC; 100 ~ 300 × 10^9^/L for PLT; 9 ~ 50 U/L for ALT; HLA-B27, Human leukocyte atigents-B27; BASDAI, Bath ankylosing spondylitis disease activity index; CRP, C-reactive protein; ESR, erythrocyte sedimentation rate; PLT, Platelet; RBC, red blood cells.

#### Blood collection and peripheral blood monocular cells (PBMCs) isolation

Blood samples were obtained by venous blood. PBMCs were isolated from AS patients and HC using Ficoll-Paque Plus (TBD science, China) following the manufacturer’s instruction under sterile conditions. The collected cells were used for cell cultures or frozen at −80°C until RNA extractions. Serum samples were stored at −80°C until cytokines were determined.

#### Recombinant human IL-37 protein

We have cloned, expressed, and purified human recombinant IL-37 protein, and confirmed its functions in our pervious study [16]. The concentrations of the protein were detected by Brandford methods, and the recombinant protein was stored at −20°C.

#### Cell culture condition

Whole PBMCs were cultured in RPMI 1640 (Hyclone, Thermo, USA) with 100 μg/ml streptomycin (Beyotime, China), 100 IU/ml penicillin and 10% Fetal Calf serum (Sijiqing, China) as culture medium in a humidified atmosphere of 5% CO_2_ at 37°C. Cells were stimulated with or without human recombinant IL-37 at various concentrations for 6 h, and then incubated further with LPS (1 μg/ml) for 4 h. Total RNAs were extracted [16], and cytokine transcriptions were analyzed by RT-PCR. To determine cytokine protein expressions in PBMCs, cells were stimulated with or without human recombinant IL-37 at 100 ng/ml for 24 h, and then incubated further with LPS (1 μg/ml) for 8 h, culture supernatants were harvested and frozen at −80°C for later cytokine analysis by ELISA.

#### RNA extraction and real-time polymerase chain reaction (RT-PCR)

Total RNAs were extracted from PBMCs using Trizol (Invitrogen, Carlsbad, CA, USA) according to the manufacturer’s protocol. The quantity and purity of RNA were then detected by Epoch 18 multi-volume Spectrophotometer System (Biotek, USA) at 260 nm and 280 nm. For subsequent reverse transcription reaction, samples with ratios from 1.8 to 2.0 were accepted. cDNAs were obtained using the iScript™ cDNA Synthesis Kit (Thermo, Pittsburgh PA, USA). RT-PCRs were performed using the SYBR Green PCR kit (Bio-rad, USA) and a CFX96 Real-Time PCR system (Bio-rad, USA). PCR products were amplified in duplicate in a total volume of 20 μL, verified by melting curve analysis. Relative mRNAs levels of target genes were calculated with normalization to β-actin values using the 2^-ΔΔct^ method. The primer sequences were summarized (Table 2).

**Table 2:** **List of the sequence of human gene primers**

**Gene name**	**Forward (5′ to 3′)**	**Reverse (5′ to 3′)**
TNF-α	ACCTCTCTCTAATCAGCCCTCT	GGGTTTGCTACAACATGGGCTA
IL-6	AGCCACTCACCTCTTCAGAAC	ACATGTCTCCTTTCTCAGGGC
IL-17	CCCGGACTGTGATGGTCAAC	GCACTTTGCCTCCCAGATCA
IL-23	GAGCAGCAACCCTGAGTCCCTA	CAAATTTCCCTTCCCATCTAATAA
IL-37	AGTGCTGCTTAGAAGACCCGG	AGAGTCCAGGACCAGTACTTTGTGA
Actin-β	CCTGACTGACTACCTCATGAAG	CGTAGCACAGCTTCTCCTTA

#### Enzyme-Linked Immunosorbent Assay (ELISA)

Serum IL-37 levels were measured with a commercially available enzyme-linked immunosorbent assay (ELISA) kit that specifically detects IL-37 (AdipoGen AG, Liestal, Switzerland). Detections of the cytokine TNF-α, IL-17, IL-6 and IL-23 were accomplished by using eBioscience ELISA Kit (San Diego, CA, USA).

#### Statistical analysis

Data were expressed as mean (± SEM) analyzed by Graphpad Prism V.5.00 software (GraphPad Software, San Diego CA, USA). Differences between two groups were performed with Mann–Whitney *U*-test for nonparametric data. Spearman correlation test was used to evaluate the associations between serum IL-37 levels and laboratory values as well as serum cytokine levels. For all experiments, P < 0.05 was considered as statistically significant.

## Results

### Elevated expression of IL-37 mRNA in PBMCs from patients with AS especially patients with active AS

**Figure 1:**
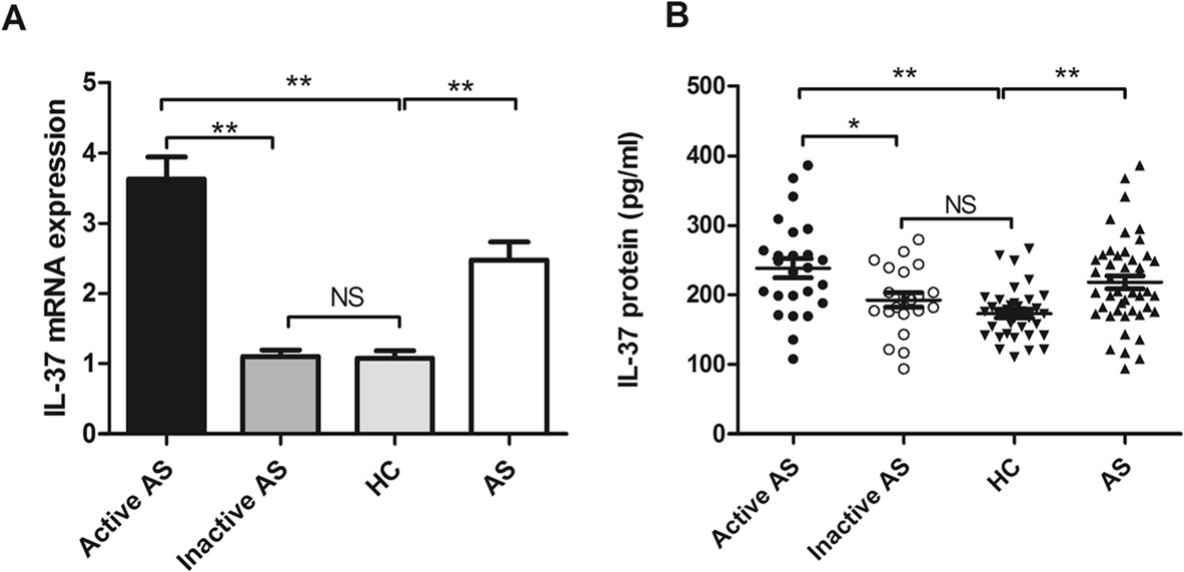
**Comparison of IL-37 mRNAs and protein levels between AS and HC. (A)** The expressions of IL-37 mRNA from PBMCs in active (n = 25) and inactive AS patients (n = 21) and in HC (n = 35) were measured by RT-PCR, results are expressed as mean ± SEM. **(B)** Serum IL-37 levels in an active (n = 25) and inactive AS patients (n = 21) and HC (n = 35) were measured by ELISA. Each symbol represents an individual AS patient and HC. Horizontal lines indicate median values. Differences between two groups were performed with Mann–Whitney *U*-test for nonparametric data. AS, ankylosing spondylitis; HC, healthy control; NS, not significant; *P < 0.05; **P < 0.01.

To investigate the association between IL-37 levels and AS, IL-37 mRNA expression in PBMCs from AS patients and healthy controls was detected by RT-PCR. Our results for the first time revealed that the expressions of IL-37 mRNA in PBMCs from AS patients were higher than those from HC (Figure 1A). We next analyzed IL-37 mRNA expressions in PBMCs from patients with active AS and inactive AS and found that a significant up-regulation of IL-37 mRNA expressions in active AS patients compared with inactive AS patients and healthy controls (Figure 1A). No difference was observed in IL-37 mRNA levels between inactive AS patients and healthy controls (Figure 1A).

### IL-37 is elevated in the serum from patients with AS especially patients with active AS

As shown in Figure 1B, serum IL-37 protein levels were significantly higher in AS patients compared with HC. Moreover, we identified a significant elevation of serum IL-37 levels in active AS patients compared with inactive AS patients and HC (Figure 1B). There is no difference in the serum IL-37 levels between patients with inactive AS and healthy controls (Figure 1B).

### Relationships between serum IL-37 levels and disease activity as well as laboratory indexes in patients with AS

**Figure 2:**
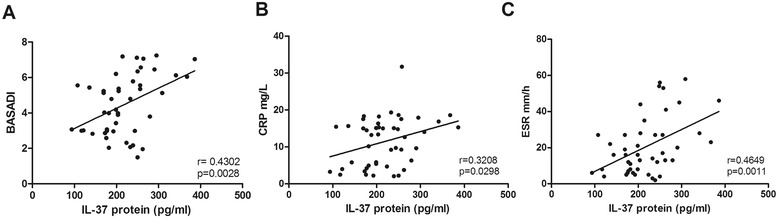
**Correlations of serum IL-37 levels and laboratory values.** Serum IL-37 levels were positively correlated with BASDAI **(A)**, CRP **(B)**, and ESR **(C)** respectively. Each symbol represents an individual patient. BASDAI, Bath Ankylosing Spondylitis Disease Activity Index. ESR, erythrocyte sedimentation rate; CRP, C-reactive protein. The correlations were evaluated with Spearman’s non-parametric test. P < 0.05 represents a significant difference.

Next, we examined the potential relationship of IL-37 levels with laboratory values including erythrocyte sedimentation rate (ESR), C-reactive protein (CRP) and clinical assessments of disease activity (defined by BASDAI). The results revealed that serum IL-37 levels were positively associated with BASDAI (Figure 2A, r = 0.4302, p = 0.0028). Accordingly, serum IL-37 levels have a significantly positive correlation with CRP (Figure 2B, r = 0.3208, p = 0.0298), and ESR (Figure 2C, r = 0.4649, p = 0.0011), respectively. But, we did not observe the correlation of serum IL-37 levels with red blood cell (RBC), alanine aminotransferase (ALT) (Table 3).

**Table 3:** **Correlation between IL-37 levels and pro-inflammatory cytokines as well as AS laboratory values**

**Parameter**	**Correlation coefficient (r)**	**P-value**
TNF-α	0.3907	0.0073
IL-6	0.4005	0.0058
IL-17	0.4922	0.0005
IL-23	0.1095	0.4689
BASADI	0.4302	0.0028
CRP	0.3208	0.0298
ESR	0.4649	0.0011
ALT	−0.2397	0.1086
PLT	0.2596	0.0815
RBC	0.04482	0.7674

BASDAI, Bath ankylosing spondylitis disease activity index; CRP, C-reactive protein; ESR, erythrocyte sedimentation rate; ALT, alanine aminotransferase; PLT, Platelet; RBC, red blood cell. The correlations were evaluated with Spearman’s non-parametric test. P < 0.05 represents a significant difference.

### Associations of serum IL-37 with pro-inflammatory cytokines levels

Published studies demonstrated that pro-inflammatory cytokines IL-17, TNF-α, IL-6 and IL-23 play an important role in promoting disease development of AS [5-11]. Consistent with these findings, we also demonstrated that the levels of serum IL-17, TNF-α, IL-6 and IL-23 were significantly higher in patients with AS than healthy controls (data not shown).

**Figure 3:**
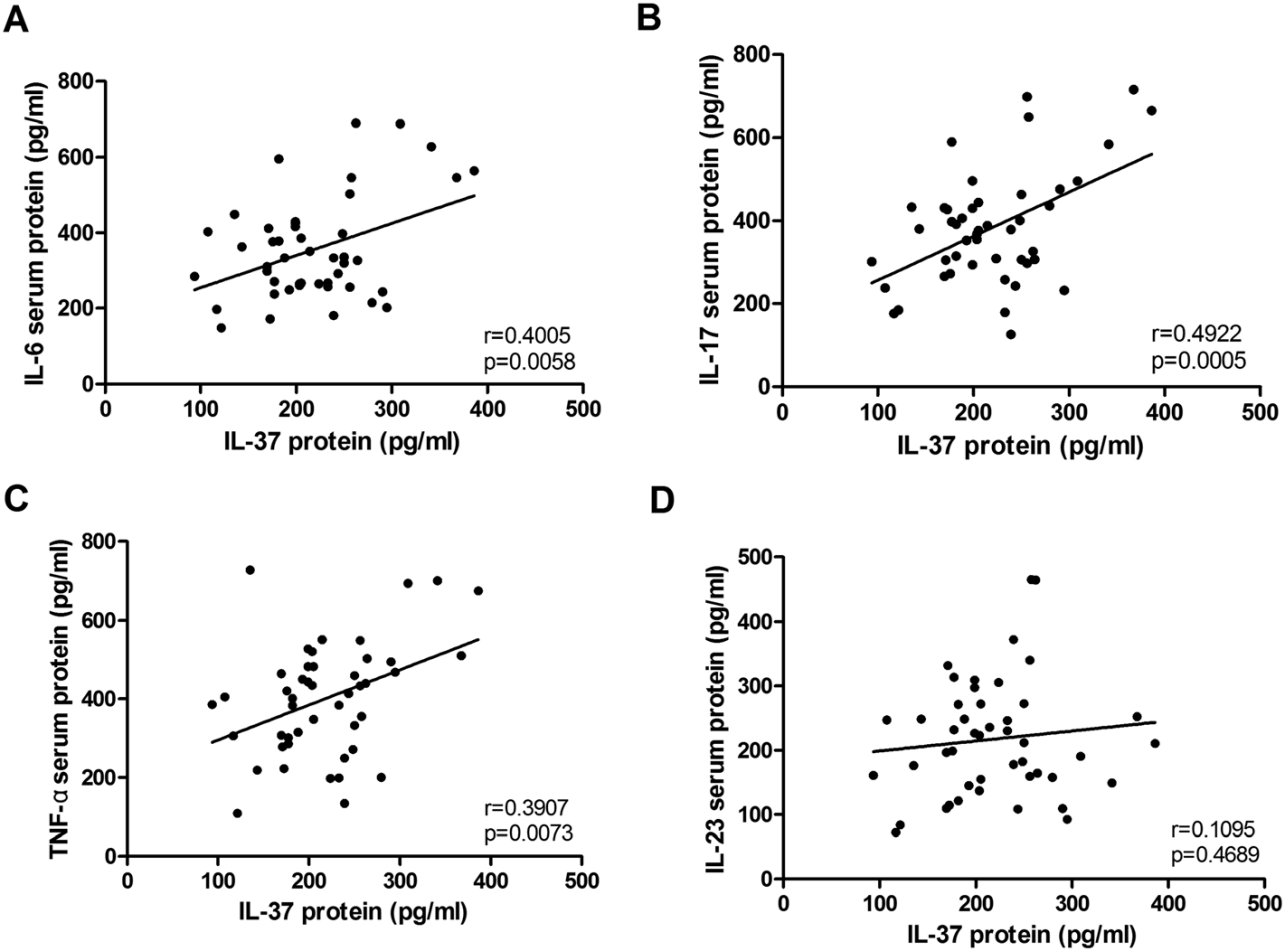
**Associations between serum levels of IL-37 and pro-inflammatory cytokines in AS patients.** Serum IL-37 levels were positively correlated with IL-6 **(A)**, IL-17 **(B)**, TNF **(C)** respectively except for IL-23 **(D)**. Each symbol represents an individual patient. The correlations were evaluated with Spearman’s non-parametric test. P < 0.05 represents a significant difference. NS, not significant.

To assess the potential relationships of serum IL-37 levels and the levels of above mentioned pro-inflammatory cytokines in patients with AS, the correlations between IL-37 and IL-6, IL-17, TNF-α, IL-23 were analyzed by Spearman correlation test. The results indicated that the concentrations of serum IL-37 levels were positively correlated with the levels of serum IL-6 (Figure 3A, r = 0.4005, P = 0.0058), IL-17 (Figure 3B, r = 0.4922, p = 0.0005) and TNF-α (Figure 3C, r = 0.3907, p = 0.0073), respectively. However, no significant correlation was observed between IL-37 and IL-23 in AS patients (Figure 3D, r = 0.1095, p = 0.4689).

### Correlations between serum IL-37 levels and AS clinical features

**Figure 4:**
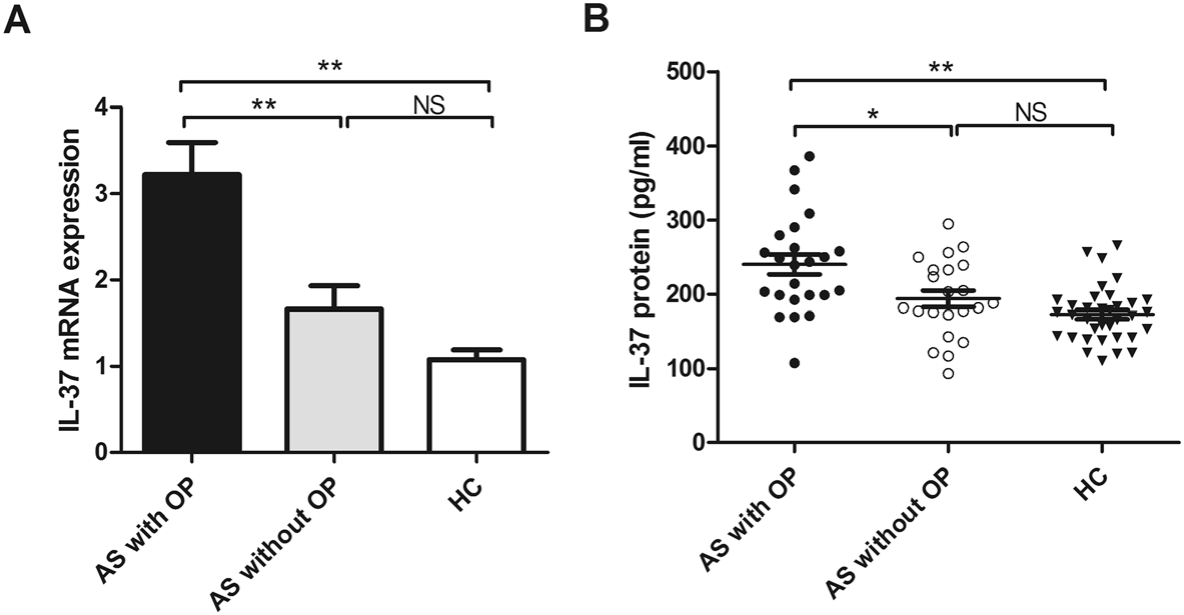
**Elevated IL-37 mRNA and protein levels in AS patients with osteoporosis and complications.** The expressions of IL-37 mRNAs in PBMCs **(A)** and IL-37 protein levels in serum **(B)** were measured in AS patients with OP (n = 24) and without OP (n = 22), as well as HC (n = 35). Results are expressed as mean ± SEM **(A)** Each individual is expressed as an symbol; horizontal lines indicate median values **(B)**. OP, osteoporosis; HC, healthy control; NS, not significant; *P < 0.05; **P < 0.01.

To further evaluate the relationships between serum IL-37 levels and AS clinical manifestations, the levels of serum IL-37 were used to compare among patients with and those without AS definite clinical features. Our data showed that no significant differences in serum IL-37 levels with peptic ulcer, liver dysfunction, intestinal tuberculosis, leukocytosis, kidney dysfunction, polycythemia, hyperlipidemia and adult still disease in AS patients (Table 4). Interestingly, the expressions of IL-37 mRNA in PBMCs (Figure 4A) and the levels of serum IL-37 (Figure 4B) were significantly higher in patients with osteoporosis compared with patients without osteoporosis. However, compared to healthy controls, AS patients without osteoporosis did not show significantly higher IL-37 mRNA (Figure 4A) and protein levels (Figure 4B). We also found that an elevated IL-37 protein level from active AS patients with OP than inactive AS patients with OP as well as HC. Similarly, compared to inactive AS patients without OP and HC, active AS patients without OP showed higher IL-37 levels in serum [Additional file 1]. These results indicated the expressions of IL-37 are closely related to the AS patients with osteoporosis.

**Table 4:** **Serum IL-37 protein levels in the presence or absence of AS clinical characteristics**

**Clinical characteristics**	**n**	**Present median (interquartile range)**	**n**	**Absent median (interquartile range)**	**P-value**
Osteoporosis	24	239.970 (386.146 to 107.470)	22	193.888 (93.536 to 294.802)	0.0263
Peptic ulcer	8	236.358 (135.338 to 341.248)	38	214.052 (93.536 to 386.146)	ns
liver dysfunction	17	225.617 (116.759 to 279.320)	29	213.832 (93.536 to 386.146)	ns
Intestinal tuberculosis	1	198.814	45	218.356 (93.536 to 386.146)	ns
Leukocytosis	7	250.126 (169.398 to 367.568)	39	212.152 (93.536 to 386.146)	ns
Kidney dysfunction	11	221.896 (107.470 to 386.146)	35	216.685 (93.536 to 341.248)	ns
polycythemia	8	246.421 (169.398 to 367.568)	38	213.114 (93.536 to 386.146)	ns
Hyperlipidemia	1	214.296	45	218.012 (93.536 to 386.146)	ns
Adult still disease	1	249.905	45	214.193 (93.536 to 367.568)	ns

Differences between two groups were performed with Mann–Whitney *U*-test for nonparametric data. P < 0.05 represents a significant difference. NS. Not significant.

### Recombinant IL-37 decreases the productions of pro-inflammatory cytokines in PBMCs from patients with AS

**Figure 5:**
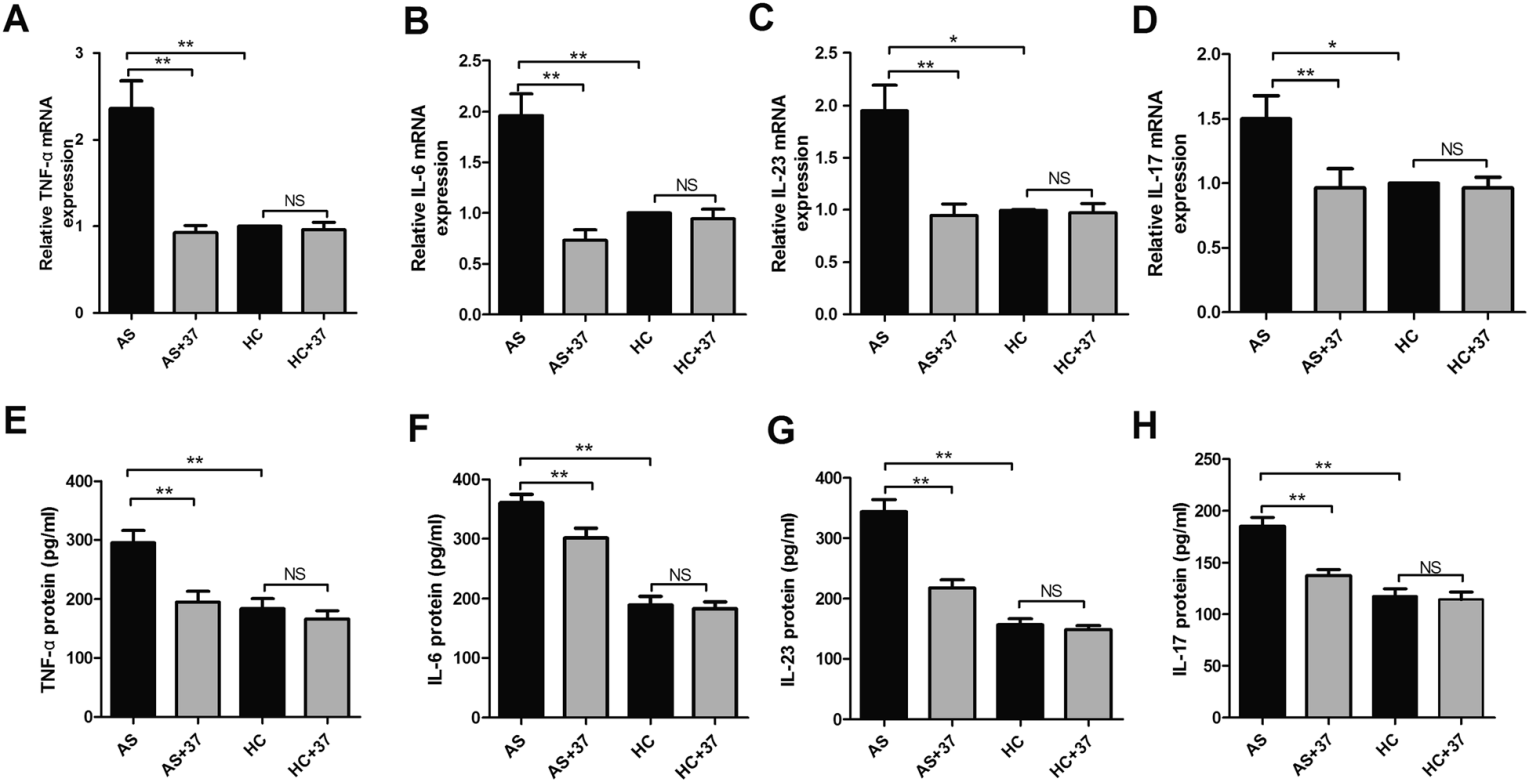
**Recombinant IL-37 decreases pro-inflammatory cytokines productions in PBMCs of AS patients.** PBMCs from AS patients (n = 46) and HC (n = 35) were stimulated with or without IL-37 (100 ng/ml) for 6 h, cells then cultured with LPS (1 ug/ml) for 4 h, the total RNAs were extracted and analyzed for TNF-α **(A)**, IL-6 **(B)** IL-23 **(C)** and IL-17 **(D)** mRNAs by RT-PCR. PBMCs from AS patients (n = 46) and HC (n = 35) were stimulated with or without IL-37 (100 ng/ml) for 24 h and then incubated further with LPS (1 μg/ml) for 8 h, supernatants of the cells were examined for TNF-α **(E)**, IL-6 **(F)**, IL-23 **(G)** and IL-17 **(H)** protein levels using ELISA. Results are expressed as mean ± SEM, P < 0.05 represents a significant difference. AS + IL-37, PBMCs from AS stimulated with the recombinant IL-37; HC + 37, PBMCs from HC stimulated with the recombinant IL-37; NS, not significant; *P < 0.05; **P < 0.01.

IL-37 has been reported to play an anti-inflammatory role in auto-immune and inflammatory disease through down-regulation of inflammatory response [16-18]. To investigate whether IL-37 has a similar capacity to decrease the expressions of pro-inflammatory cytokines involved in the pathogenesis of AS. First of all, we had expressed and purified recombinant human IL-37 protein based on previous methods [16]. To determine the anti-inflammatory role of IL-37 in AS, the PBMCs isolated from AS patients and HC were cultured in the presence or absent of recombinant IL-37 and further with LPS stimulation. The cells and cultural supernatants were harvested for ELISA and RT-PCR analysis, respectively. We found that the expressions of pro-inflammatory cytokines TNF-α (Figure 5A), IL-6 (Figure 5B), IL-23 (Figure 5C) and IL-17 (Figure 5D) mRNA in the PBMCs of AS patients were dramatically suppressed by IL-37 treatment. Meanwhile, IL-37 also markedly reduced the secretions of TNF-α (Figure 5E), IL-6 (Figure 5F), IL-23 (Figure 5G) and IL-17 (Figure 5H) in the cultural supernatants of the PBMCs in AS patients. Interestingly, the productions of these cytokines mRNAs and protein levels of the PBMCs in HC were unaffected by IL-37 stimulation (Figure 5A-H).

## Discussion

Our previous studies have demonstrated that IL-37 plays as an immune mediator to restrain the inflammatory response of autoimmune diseases in SLE and GD [16,30]. Other published data have shown that IL-37 has a pivotal function in inflammatory regulation of inflammatory disorders [17,18]. Yet, it is still not clear whether IL-37 is involved in the pathogenesis of AS. Here, we for the first time reported that IL-37 concentrations in serum and IL-37 mRNA expression in PBMCs were dramatically higher in AS patients compared to HC, and the levels of IL-37 positively correlated with the disease activity, especially with the major pro-inflammatory cytokines related to AS. These data suggested IL-37 closely relates to AS, especially the activity of AS, and may play an important role in inflammatory regulation within the AS.

Several complications often present in AS patients, such as uveitis, inflammatory bowel disease, and psoriasis [4]. Osteoporosis (OP) is a common complication in the pathogenesis of AS and it can raise the risk of bone fracture [34]. The published data have indicated that osteoporosis was related to the AS disease activity [35]. In this study, our experiments showed that OP was more common in active AS patients than in inactive AS patients (Table 1). Pro-inflammatory cytokines have been shown to be involved in AS disease activity [8,36]. Marina Magrey et al. revealed that OP was associated with the high levels of pro-inflammatory cytokines in AS patients [37]. Indeed, our results confirmed that serum pro-inflammatory cytokines levels were significantly higher in AS patients with OP than those without OP. Interestingly, our experiments also indicated that the levels of serum IL-37 were significantly higher in AS patients with OP than those without OP (Figure 4). Furthermore, we observed the positively correlation between the levels of IL-37 and several useful markers of the disease activity such as BASDAI, CRP and ESR. These results consisted with our published data that inflammatory reaction stimulates IL-37 production [16,30]. Taken together, our result implies that AS inflammatory reaction and their inflammatory cytokines might play a positive feedback to induce the expression of IL-37 during the complication development of AS.

Although the mechanism of pathogenesis in AS is poorly understood, the published data have indicated that pro-inflammatory cytokines play an important role in the inflammatory development in AS [6-12]. Previous studies demonstrated that serum levels of TNF, IL-6, IL-23 and IL-17 are significantly elevated in AS patients and blocking these pro-inflammatory cytokines with antibodies or recombinant soluble receptor dramatically alleviated axial and peripheral inflammation in AS [13-15]. In this study, we observed the higher serum levels of these cytokines in AS than that in HC (data not shown). More importantly, we also found that the elevating levels of IL-37 in serum and PBMCs from patients with AS compared to HC. Spearman correlation test analysis also showed that serum IL-37 levels were positively correlated with major pro-inflammatory cytokines such as TNF, IL-6 and IL-17 in AS. It has been demonstrated that the IL-37 mRNA and protein expression can be induced by TNF-α via activation of nuclear factor(NF)-kB and activator protein (AP)-1 signaling pathway in intestinal epithelial cell [38]. Similarly, TNF-α also induce IL-37 expression in PBMCs from healthy controls [22], indicating TNF-α as one of inducers involved in the up-regulation of IL-37 in AS. Not only that, using anti-TNF-α antibody reduces the levels of IL-6 and Th17 responses, which reflects that IL-6 and other cytokines serve as an important downstream effectors of TNF-α pathway [14,39]. Thus, we speculate that inflammation signaling caused by TNF-α signal pathway not only exacerbated the inflammatory response in pathogenesis of AS but also promoted the expression of anti-inflammatory cytokine IL-37 to limit excessive inflammation in AS. However, further studies are needed to define the direct or indirect role of these cytokines in driving IL-37 expression.

In recent reports, IL-37 was shown to be a negative mediator to reduce pro-inflammatory cytokine productions in inflammatory diseases [24,25]. To further explore the effects of IL-37 on the pro-inflammatory cytokines that are responsible for the pathogenesis of AS, the recombinant human IL-37 protein was used to stimulate PBMCs from AS patients and HC. In our study, we showed the recombinant IL-37 proteins remarkably attenuate LPS-induced TNF-α, IL-6, IL-17, IL-23 expressions in PBMCs from AS patients but not in HCs. Although the inhibitions of pro-inflammatory cytokine expression signaling pathways by IL-37 remain to elusive, Nold et al. revealed that IL-37 can reduce the expression of STAT3 [22], while the STAT3 have been reported to closely related to AS [40,41]. We suspect that IL-37 may through regulate several critical signal transducers like STAT3 to attenuates the pro-inflammatory cytokines productions which may be able to improve inflammatory reactions of AS.

The findings of our study provide a new insight for unraveling the intriguing balance between IL-37 and the pro-inflammatory cytokines in AS. However, further studies are needed to determine the regulatory mechanism of IL-37 in pathogenesis of AS.

## Conclusions

Altogether, we highlighted that the levels of IL-37 are increased in AS patients, and it is associated with the pro-inflammatory cytokines, disease activity and complications. We also demonstrated that IL-37 can effectively alleviate the expressions of pro-inflammatory cytokines (TNF-α, IL-6, IL-17 and IL-23) that are responsible for the pathogenic development of AS. Hence, our study implied that the inflammatory reactions and their inflammatory cytokines in AS enhance the expression of IL-37, but IL-37 act as a negative regulator to smother the excessive inflammation, thereby protecting against OP in AS. This study might offer a new target (IL-37) for AS therapy. Further researches are necessary to identify the mechanisms and signal pathways for IL-37 functions and expression regulations in the pathogenesis of AS.

## Additional file

Additional file 1: Figure S1. IL-37 protein levels in active and inactive AS patients with or without OP as well as HC. **(A)** IL-37 protein levels in serum were measured in active (n = 7) and inactive (n = 15) AS patients without OP as well as HC (n = 35). **(B)** IL-37 protein levels in serum were measured in active (n = 18) and inactive (n = 6) AS patients with OP as well as HC (n = 35). Results are expressed as mean ± SEM. Each individual is expressed as an symbol; horizontal lines indicate median values. OP, osteoporosis; HC, healthy control; NS, not significant; *P < 0.05; **P < 0.01.

## Abbreviations

AS: Ankylosing spondylitis
OP: Osteoporosis
IL-37: Interleukin-37
MHC I: Major histocompatibility complex class I
LPS: Lipopolysaccharide
TNF-α: Tumor necrosis factor-α
IL-17: Interleukin-17
IL-6: Interleukin-6
IL-23: Interleukin-23
HC: Healthy controls
HLA-B27: Human leukocyte atigents-B27
PBMCs: Peripheral blood mononuclear cells
qRT-PCR: Real-time quantitative PCR
ELISA: Enzyme-linked immunoassay
BASDAI: Bath ankylosing spondylitis disease activity index
SLE: System lupus erythematosus
RA: Rheumatoid arthritis
GD: Graves’ disease
CRP: C-reactive protein
ESR: Erythrocyte sedimentation rate
PLT: Platelet
RBC: Red blood cell
ALT: Alanine aminotransferase
