# Supplementary figures and images for: Interleukin-37 is increased in ankylosing spondylitis patients and associated with disease activity
Source: J Transl Med. 2015 Jan 28;13:36. doi: 10.1186/s12967-015-0394-3 (PMC4323018; doi:10.1186/s12967-015-0394-3)

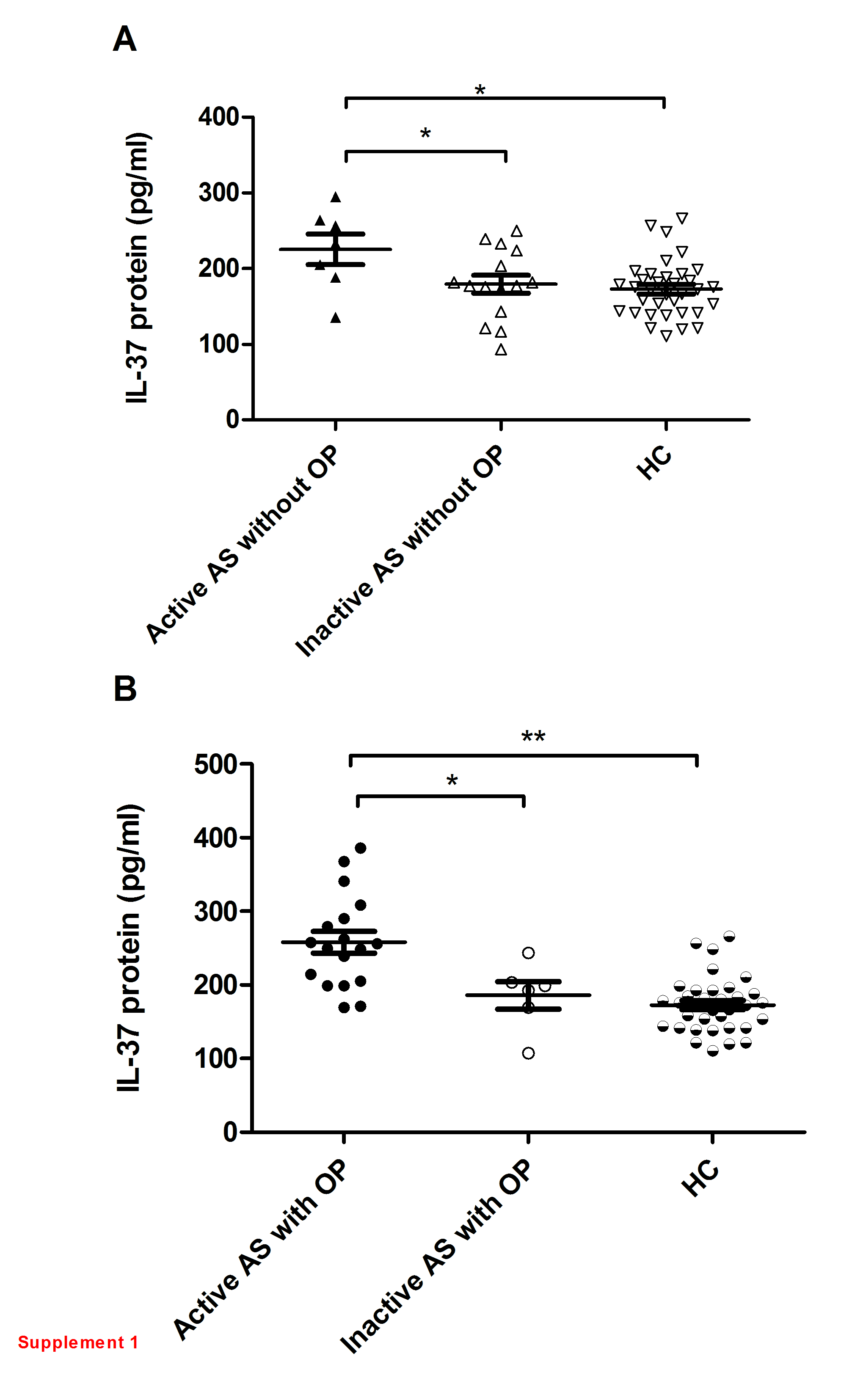

Supplement: Additional file 1: Figure S1. — IL-37 protein levels in active and inactive AS patients with or without OP as well as HC. (A) IL-37 protein levels in serum were measured in active (n = 7) and inactive (n = 15) AS patients without OP as well as HC (n = 35). (B) IL-37 protein levels in serum were measured in active (n = 18) and inactive (n = 6) AS patients with OP as well as HC (n = 35). Results are expressed as mean ± SEM. Each individual is expressed as an symbol; horizontal lines indicate median values. OP, osteoporosis; HC, healthy control; NS, not significant; *P < 0.05; **P < 0.01. [file 12967_2015_394_MOESM1_ESM.tiff]
